# Dexamethasone Inhibits Synergistic Induction of PDE4B Expression by Roflumilast and Bacterium NTHi

**DOI:** 10.3390/ijms19113511

**Published:** 2018-11-08

**Authors:** Byung-Cheol Lee, Seiko Susuki-Miyata, Chen Yan, Jian-Dong Li

**Affiliations:** 1 Center for Inflammation, Immunity & Infection, Institute for Biomedical Sciences, Georgia State University, Atlanta, GA 30303, USA; microlbc@korea.ac.kr (B.-C.L.); se.miyata@ono.co.jp (S.S.-M.); 2 Aab Cardiovascular Research Institute and Department of Medicine, University of Rochester Medical Center, 601 Elmwood Avenue, Rochester, NY 14642, USA; chen_yan@urmc.rochester.edu

**Keywords:** dexamethasone, PDE4B, roflumilast, nontypeable *Haemophilus influenzae*, glucocorticoid receptor

## Abstract

Phosphodiesterase 4B (PDE4B) plays an important role in inflammation. Recently we have reported that roflumilast as a PDE4-selective inhibitor, synergizes with nontypeable *Haemophilus influenzae* (NTHi) to up-regulate PDE4B expression in vitro and in vivo. Clinical evidence and our previous results suggest that synergistic induction of PDE4B could be counterproductive for suppressing inflammation or may contribute to tolerance to roflumilast. We thus investigated if dexamethasone inhibits the synergistic induction of PDE4B by roflumilast and NTHi as well as inflammation. Here, dexamethasone markedly suppressed the synergistic induction of PDE4B in human lung epithelial cells and in vivo. We also found that dexamethasone further suppressed NTHi-induced inflammatory response in vitro and in vivo. Moreover, Compound A, as a dissociating non-steroidal glucocorticoid receptor (GR) ligand, inhibited the synergistic induction of PDE4B, thereby suggesting the requirement of dexamethasone-mediated GR activation in the suppression of PDE4B expression. Taken together, our data suggest that dexamethasone may help attenuate inflammation and tolerance through suppressing the PDE4B expression in chronic obstructive pulmonary disease (COPD) patients using roflumilast.

## 1. Introduction

Intracellular cAMP is inactivated by the phosphodiesterases (PDEs), leading to the suppression of inflammatory responses [1,2,3,4]. Each identified PDE (PDE1-11) has several isoforms and transcriptional/splice variants with distinct properties [5,6,7]. In particular, PDE4 is a major therapeutic target protein in chronic obstructive pulmonary disease (COPD), asthma, and cystic fibrosis (CF) [8,9,10]. Previous studies showed that cigarette smoke extracts, cAMP elevator, lipopolysaccharide (LPS), and nontypeable *Haemophilus influenzae* (NTHi) induced expression of PDE4, pro-inflammatory cytokines and chemokines in various cell types, including airway epithelial cells and leukocytes [11,12,13,14,15,16,17].

Roflumilast, a second generation PDE4-selective inhibitor, was approved in 2012 for treating severe COPD patients with exacerbations and chronic bronchitis [18,19,20]. In addition, roflumilast has been shown to inhibit a broad range of inflammatory cytokines and chemokines in human neutrophils and pulmonary epithelial cells [21,22,23]. However, clinical evidence suggests that treatment with repeated dosing of roflumilast may lead to the development of tachyphylaxis or tolerance for roflumilast through up-regulation of phosphodiesterase 4B (PDE4B) expression [24,25,26,27]. Recently, we have reported that roflumilast synergizes with NTHi to induce the pro-inflammatory cytokines and chemokines through upregulation of PDE4B expression in vitro and in vivo [28]. Thus, understanding how up-regulation of PDE4B expression by roflumilast and NTHi is attenuated may help to improve the efficacy of roflumilast.

Glucocorticoids (GCs) are the most widely used agents for controlling inflammatory diseases such as COPD and asthma [23,29,30,31,32]. GCs directly bind to glucocorticoid receptors (GRs) and are then translocated to the nucleus, thereby suppressing the activity of nuclear factor-κB (NF-κB) and activator protein 1 (AP-1) and leading to decreased inflammatory gene expression [33,34]. Recent studies also showed that dexamethasone (Dex) decreases PDE4B expression in human pulmonary endothelial cells and osteosarcoma cells [32,35]. Thus, we hypothesized that dexamethasone may suppress the synergistic induction of PDE4B by roflumilast and NTHi and improve the anti-inflammatory effects and the side effects of roflumilast through down-regulation of PDE4B expression.

## 2. Results

### 2.1. Dexamethasone Suppresses Synergistic Induction of PDE4B Expression by Roflumilast and NTHi In Vitro and In Vivo

**Figure 1:**
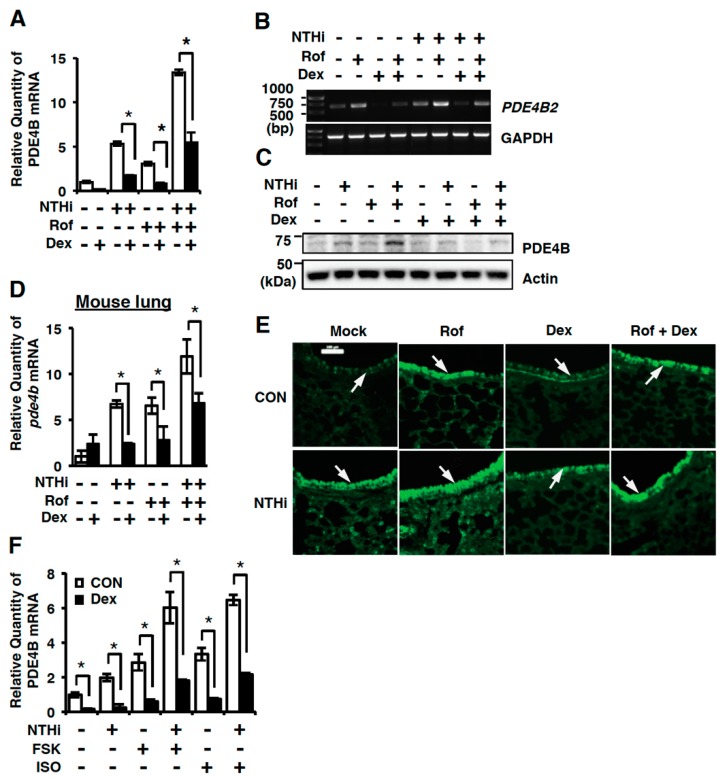
Dexamethasone suppresses up-regulation of phosphodiesterase 4B (PDE4B) expression by roflumilast and NTHi in vitro and in vivo. (**A**) BEAS-2B cells were pretreated with Roflumilast (Rof) (0.1 µM) and dexamethasone (Dex) (10 nM) for 1 h followed by 1.5 h stimulation with NTHi, and *PDE4B* mRNA expression was analyzed by Q-PCR. Data are mean ± S.D. (*n* = 3); * *p* < 0.05. (**B**) BEAS-2B cells were pre-treated with Rof (0.1 µM) and dexamethasone (Dex) (10 nM) for 1 h followed by 1.5 h stimulation with NTHi, and *PDE4B2* mRNA expression was analyzed by semi-quantitative RT-PCR. (**C**) BEAS-2B cells were pre-treated with Rof (0.1 µM) and Dex (10 nM) for 1 h followed by 5 h stimulation with NTHi, and PDE4B protein expression was analyzed by western blot. (**D**,**E**) Mice were inoculated with Rof (5 mg/kg i.p.) and/or Dex (2 mg/kg i.p.) for 2 h, followed by intratracheal inoculation with NTHi (5 × 10^7^ CFU/lung). (**D**) After 5 h, mRNA expression in lung tissues was analyzed by Q-PCR. Data are mean ± S.D. (*n* = 3); * *p* < 0.05. (**E**) Lung tissues were stained against PDE4B (Magnification ×200, Scale bar, 100 μm). Arrows indicate bronchial epithelium of mouse lung tissues. (**F**) BEAS-2B cells were pre-treated with forskolin (FSK) (1 µM), Isoproterenol (ISO) (1 µM) and Dex (10 nM) for 1 h followed by 1.5 h stimulation with NTHi, and mRNA expression was analyzed by Q-PCR. Data are mean ± S.D. (*n* = 3); * *p* < 0.05. All the relative quantity of mRNA is relative to a house keeping gene *cyclophilin* or *GAPDH*. Data are representative of three independent experiments.

We first sought to determine if dexamethasone suppresses the synergistic induction of PDE4B and thereby improves its efficacy in human bronchial epithelial BEAS-2B cells by performing quantitative PCR (Q-PCR), semi-quantitative RT-PCR analysis (RT-PCR), and western blot analysis. As shown in Figure 1, dexamethasone markedly inhibited induction of PDE4B induced by either NTHi or roflumilast alone or both in BEAS-2B cells (Figure 1A–C). Consistent with in vitro results, dexamethasone also suppressed the synergistic induction of *pde4b* expression at mRNA level in mouse lung (Figure 1D). Similar result was also observed by performing immunofluorescent staining in the mouse lung (Figure 1E). To further investigate the effects of dexamethasone on cAMP-induced PDE4B expression, BEAS-2B cells were pre-treated with cAMP inducer, such as forskolin and isoproterenol, or dexamethasone for 1h followed by 5 h stimulation with NTHi. Dexamethasone significantly inhibited the *PDE4B* induction in BEAS-2B cells (Figure 1F). Together, our data suggest that dexamethasone suppresses the synergistic induction of PDE4B expression by roflumilast and NTHi at mRNA and protein levels in vitro and in vivo.

### 2.2. Dexamethasone Suppresses NTHi-Induced Inflammation In Vitro and Mouse Lung In Vivo

**Figure 2:**
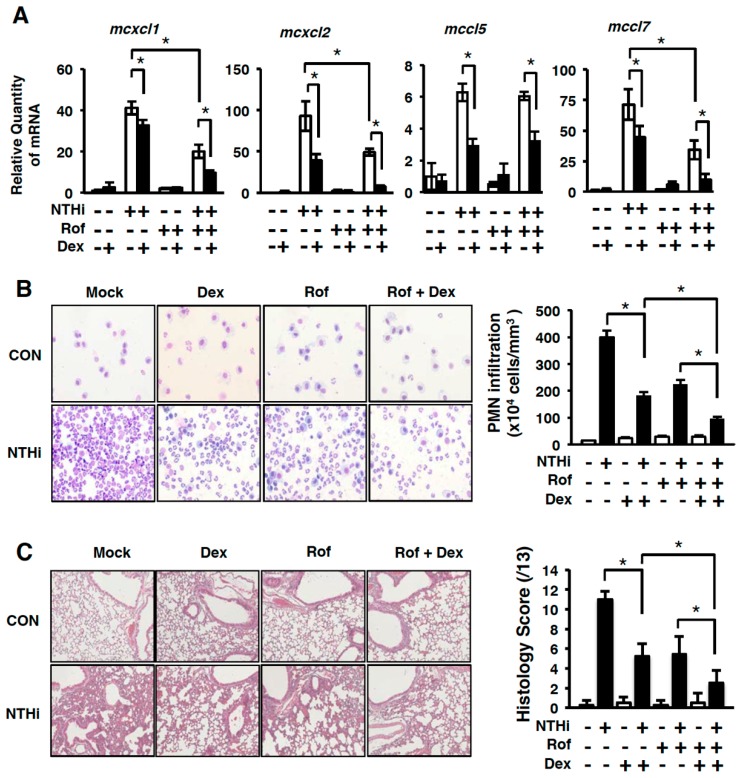
Dexamethasone suppresses NTHi-induced infiltration of polymorphonuclear neutrophils (PMNs) and inflammation in mouse lung in vivo. (**A**) Mice were inoculated with Rof (5 mg/kg i.p.) and/or Dex (2 mg/kg i.p.) for 2 h, followed by intratracheal inoculation with NTHi (5 × 10^7^ CFU/lung). After 5 h, mRNA expressions of pro-inflammatory mediators in lung tissues were analyzed by Q-PCR. The relative quantity of mRNA is relative to a house keeping gene *GAPDH*. Data in (**A**) are mean ± S.D. (*n* = 3); * *p* < 0.05. (**B**) Mice were inoculated with Rof (5 mg/kg i.p.) and/or Dex (2 mg/kg i.p.) for 2 h, followed by intratracheal inoculation with NTHi (5 × 10^7^ CFU/lung) for 9 h. Bronchoalveolar lavage (BAL) fluid was harvested and cells from BAL fluid were then cytocentrifuged and stained with Diff-Quik staining kit. (Magnification: 100×). The number of PMN cells from BAL fluid was counted by using a hemocytometer under the microscope. Data are mean ± S.D. (*n* = 3); * *p* < 0.05. (**C**) The lung tissues were collected 9 h after NTHi inoculation. Lung tissues were fixed with 10 % formalin, embedded with paraffin, and H&E stained (Magnification: 100×). Data are mean ± S.D. (*n* = 3); * *p* < 0.05. All data are representative of three independent experiments.

The pro-inflammatory mediators including cytokines and chemokines play a critical role in airway inflammatory diseases, such as COPD, through the recruitment and activation of leukocytes from the circulation to the lung [17]. Previously we reported that PDE4B2 is critical for mediating NTHi-induced chemokines in both a PDE enzymatic activity-dependent and -independent manner [28]. Thus, we examined the effects of dexamethasone on the induction of chemokines induced by either NTHi or roflumilast alone, or both, in mouse lungs by performing Q-PCR analyses. As shown in Figure 2A, dexamethasone markedly inhibited the expression of *cxcl1*, *cxcl2*, *ccl5* and *ccl7* in the mouse lungs.

To further determine the effects of dexamethasone on NTHi-induced inflammation in the lungs of mice, we performed hematoxylin and eosin (H&E) staining and evaluated the recruitment of polymorphonuclear neutrophils (PMN) in bronchoalveolar lavage (BAL) fluid. NTHi induced recruitment of PMNs. The recruitment of PMN in BAL fluid was inhibited by roflumilast or dexamethasone treatment in mouse lungs. Interestingly, recruitment of PMN was further inhibited by treatment with both roflumilast and dexamethasone (Figure 2B). Similar results were also confirmed by performing histological analysis (H&E staining) to evaluate the effects of dexamethasone on the NTHi-induced inflammation in the lungs of mice (Figure 2C). Thus, we suggest that dexamethasone-induced inhibition of chemokine expression leads to the suppression of inflammation and PMN infiltration.

### 2.3. Dexamethasone Improves the Inhibitory Effect of Roflumilast on NTHi-Induced Inflammation by Suppressing Up-Regulated PDE4B

**Figure 3:**
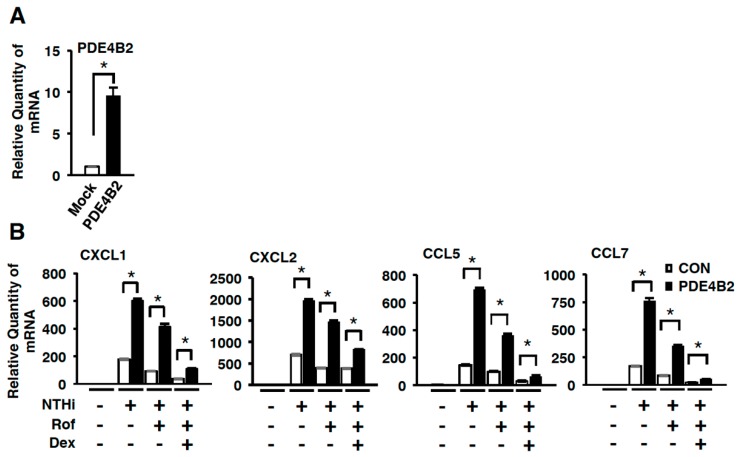
Dexamethasone improves the anti-inflammatory effect of roflumilast in cells stably expressing PDE4B. (**A**) *PDE4B* expression in mock and PDE4B2-stable cells. Data are mean ± S.D. (*n* = 3); * *p* < 0.05. (**B**) Mock or PDE4B2 stable cells were pre-treated with Rof (10 µM) or Dex (100 nM) for 1 h followed by 5 h stimulation with NTHi, and mRNA expression of pro-inflammatory mediators was analyzed by Q-PCR. The relative quantity of mRNA is relative to a house keeping gene *cyclophilin*. Data are mean ± SD (*n* = 3); * *p* < 0.05. Data are representative of three independent experiments.

We next sought to determine if dexamethasone indeed suppresses the NTHi-induced inflammatory response mediated through up-regulated expression of PDE4B, by developing and using BEAS-2B cells stably overexpressing wild-type PDE4B2 (PDE4B2-stable cells). As shown in Figure 3A, the *PDE4B2* expression was significantly increased in PDE4B2-stable cells compared to mock cells. NTHi-induced expression of *CXCL1*, *CXCL2*, *CCL5*, and *CCL7* was significantly enhanced in PDE4B2-stable cells compared with mock cells. The increased expression of *CXCL1*, *CXCL2*, *CCL5*, and *CCL7* was not fully suppressed by roflumilast even at 10 µM, but was fully suppressed by additional treatment with dexamethasone (Figure 3B). These data suggest that dexamethasone may inhibit induction of CXCL1, CXCL2, CCL5, and CCL7 via PDE4B-dependent mechanism.

### 2.4. Dexamethasone Suppresses PDE4B Expression in a GR-Dependent Manner

**Figure 4:**
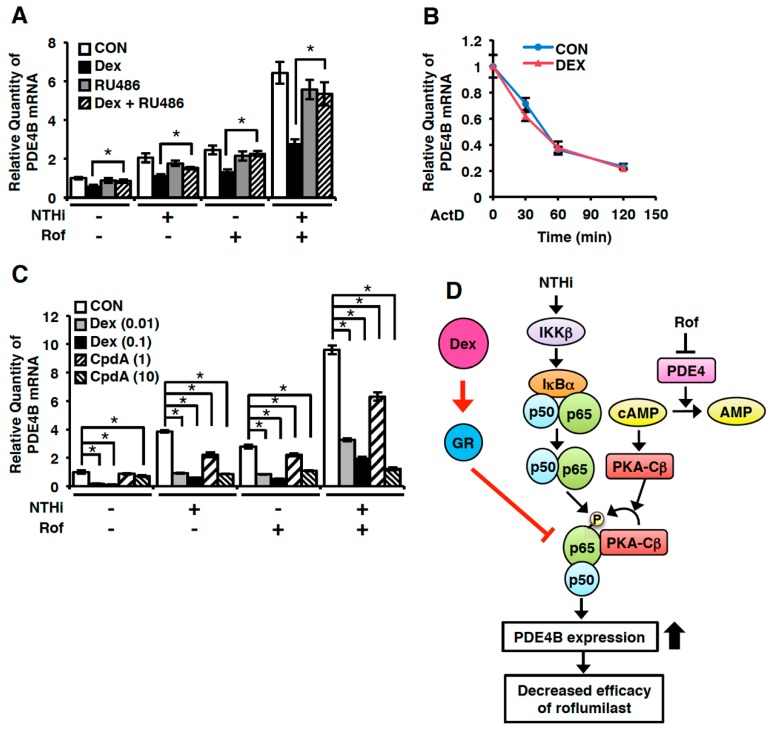
Dexamethasone suppresses PDE4B expression in a glucocorticoid receptor (GR)-dependent manner. (**A**) Rof (0.1 µM) and Dex (0.1 µM) were pre-treated with or without RU486 (1 µM) for 1 h in BEAS-2B cells, followed by 1.5 h stimulation with NTHi and *PDE4B* mRNA expression was analyzed by Q-PCR. Data are mean ± S.D. (*n* = 3); * *p* < 0.05. (**B**) ActD (5 ng/mL) was treated with or without Dex (0.1 µM) for indicated time in BEAS-2B cells. *PDE4B* mRNA expression was analyzed by Q-PCR. (**C**) BEAS-2B cells were pre-treated with Rof (0.1 µM), Dex (0.01 and 0.1 µM) and CpdA (1 and 10 µM) for 1 h followed by 1.5 h stimulation with NTHi, and *PDE4B* mRNA expression was analyzed by Q-PCR. Data are mean ± S.D. (*n* = 3); * *p* < 0.05. All the relative quantity of mRNA is relative to a house keeping gene cyclophilin. Data are representative of three independent experiments. (**D**) Schematic representation of dexamethasone-mediated suppression of PDE4B induction by roflumilast and NTHi. Arrows indicate induction or activation, and T bars indicate inhibition.

Dexamethasone regulates specific gene expression via GR-dependent and -independent mechanisms [34,36,37]. To investigate the mechanism of dexamethasone-mediated PDE4B suppression, we determined the effect of RU486 (mifepristone), as a GR antagonist, on the PDE4B expression. In the presence of RU486, the suppressive effect of dexamethasone on PDE4B expression was completely abolished, indicating that dexamethasone-mediated GR activation is required to suppress *PDE4B* expression (Figure 4A). GR activation has been shown to reduce specific gene expression either by transcriptional regulation or via post-transcriptional and non-genomic actions including pathways controlling mRNA stability [38,39]. Thus, we examined the effect of dexamethasone on the *PDE4B* mRNA stability. BEAS-2B cells were treated with Actinomycin D (ActD) in the presence or absence of dexamethasone, and the disappearance of *PDE4B* mRNA over time was quantified by Q-PCR. As shown in Figure 4B, *PDE4B* mRNA level was decreased time-dependently, which was not affected by dexamethasone, suggesting that dexamethasone suppresses PDE4B expression transcriptionally. To further investigate whether dimerization of GR is required for the PDE4B suppression, we examined the effect of Compound A (CpdA), as a GR modulator through directly binding to GR, on the PDE4B expression. Interestingly, like dexamethasone, CpdA significantly attenuated the *PDE4B* induction by NTHi and roflumilast, although the required concentration was 100-fold higher than dexamethasone (Figure 4C). Collectively, these results suggest that the dexamethasone-mediated suppression of PDE4B is GR-dependent, but likely GR dimerization-independent.

## 3. Discussion

Roflumilast is a recently approved drug for the treatment of COPD with exacerbations and chronic bronchitis. Inhibition of the enzymatic activity of PDE4B is a well-known strategy to ameliorate the symptoms [14,15,16,28,40]. However, our previous study and clinical evidence indicate that roflumilast also increases the expression of PDE4B. Induction of PDE4B could be counterproductive for suppressing inflammation and may contribute to tolerance to roflumilast in enzymatic-dependent and -independent manner [28]. The clinical efficacy of roflumilast in treating COPD may be thus compromised. Based on these interesting observations, we hypothesized that suppressing the expression of PDE4B may provide therapeutic benefit. In the present study, we have demonstrated that glucocorticoids (GCs) suppressed PDE4B expression. GCs are the most widely used agent for controlling inflammation in a number of diseases including COPD [23,29,30,31,32]. Previous studies indicate that dexamethasone decreases PDE4B expression in human pulmonary endothelial cells, airway epithelial cells, and osteosarcoma cells [32,35]. The anti-inflammatory effect exerted by increasing cAMP using roflumilast, forskolin, and isoproterenol (β-adrenoceptor) has been well demonstrated. In addition, the superior therapeutic benefits from the combination therapies of dexamethasone with PDE4 inhibitors or β2-agonists have also been reported in various studies [19,41,42]. Moreover, dexamethasone (Dex) inhibited the cigarette smoke extract (CSE)-induced expression of PDE4 in human pulmonary artery endothelial cells (HPAECs) [32]. Dex also suppressed the expression of PDE4B in human osteosarcoma cells. Consistent with these results, forskolin (FSK) and isoproterenol (ISO), the known cAMP elevators, increased the expression of PDE4B [35]. We previously reported that roflumilast synergizes with NTHi to up-regulate PDE4B expression in vitro and in vivo. In this study, we showed that treatment with Dex decreased the up-regulated PDE4B expression by NTHi and Rof or FSK and ISO stimulation (Figure 1). These results suggest that roflumilast or other cAMP elevators may become more efficacious if used in combination with Dex. The combination treatment with Dex and selective cAMP elevators is thus therapeutically desirable for treating patients with COPD and asthma.

Proinflammatory mediators including cytokines and chemokines play an important role in the activation and recruitment of leukocytes in pulmonary diseases [43,44,45]. We recently reported that NTHi significantly up-regulated the expression of chemokines including CCL5, CCL7, CXCL10, CXCL11, and IL-8. PDE4B inhibitor roflumilast markedly suppressed the induction of chemokines including CCL5 and CCL7 [28]. In addition, glucocorticoids inhibited the production of chemokines [46]. PDE4 inhibitors have been shown to reduce chemotaxis of lymphocytes and PMN through suppression of chemokine production [28,47]. In line with these findings, our results showed that the treatment with both roflumilast and Dex inhibited the expression of chemokines including CXCL1, CXCL2, CCL5, and CCL7 (Figure 2 and Figure 3).

Several studies also demonstrated additive anti-inflammatory effect of the roflumilast together with GCs in peripheral blood mononuclear cells (PBMCs), bronchial epithelial cells and CD8 lymphocytes [22,23,48]. Moreover, the REACT clinical trial study showed that treatment with roflumilast together with GCs reduced the exacerbation rates in COPD patients with a history of exacerbations [48,49]. Our histological results suggested that the combination treatment of roflumilast and dexamethasone markedly inhibited the inflammation likely through the suppression of chemokines induction and PMN infiltration in mouse lungs (Figure 2), which is consistent with previous studies showing the anti-inflammatory effects of roflumilast and glucocorticoid in vivo [28,50,51].

It is still largely unclear how Dex down-regulates PDE4B expression. Dex regulates the expression of a variety of genes via GR-dependent and –independent mechanism [34,37,52]. Recently, we have shown that roflumilast synergizes with NTHi to induce PDE4B expression through a cross-talk between NF-κB p65 and PKA-Cβ [28]. Negative cross-talk between GR and NF-κB has also been reported [53,54]. PDE4 inhibitors enhanced the action of glucocorticoids through the induction of glucocorticoid receptor (GR) expression in a dose-dependent manner [55,56]. In this study, we found that Dex significantly inhibited the expression of PDE4B induced by roflumilast and NTHi through a GR-dependent mechanism (Figure 4). Moreover, PDE4B expression is attenuated by Dex likely via the GR dimerization-independent manner (Figure 4C). Collectively, we provided supportive evidence that the addition of Dex may improve the compromised anti-inflammatory effects of Roflumilast due to the up-regulated PDE4B and reduce the side effect of roflumilast through down-regulation of PDE4B expression in a GR-dependent manner in pulmonary diseases such as COPD and asthma (Figure 4D). Therefore, our study may be helpful for developing a combination therapeutic strategy in order to improve the efficacy of roflumilast.

## 4. Materials and Methods

### 4.1. Reagents and Antibodies

Dexamethasone, RU-486 (Mifepristone), Isoproterenol, and Actinomycin D were purchased from Sigma-Aldrich (St. Louis, MO, USA). Roflumilast was purchased from Santa Cruz Biotechnology (Dallas, TX, USA). Forskolin and Compound A were purchased from Enzo Life Science (Farmingdale, NY, USA). Antibodies for β-actin (sc-8432) and PDE4B (sc-25812) were purchased from Santa Cruz Biotechnology (Dallas, TX, USA).

### 4.2. Bacterial Strains and Culture Condition

Nontypeable *Haemophilus influenzae* (NTHi) strain 12 was a clinical isolate for this study [57]. Bacteria were cultured as described previously [58] and then bacteria were washed and suspended in DMEM for in vitro cell experiments and in saline for in vivo animal experiments.

### 4.3. Cell Culture

Human bronchial epithelial BEAS-2B and PDE4B2-stable cells were maintained as described previously [28]. To investigate the effects of dexamethasone, BEAS-2B cells were pre-treated with cAMP elevators, such as forskolin (1 µM) and isoproterenol (1 µM), or dexamethasone (10 nM) for 1 h followed by 5 h stimulation with NTHi and then the expression PDE4B mRNA was analyzed by Q-PCR. To examine the mechanism of dexamethasone-mediated PDE4B suppression, Rof (0.1 µM) and Dex (0.01 and 0.1 µM) were pre-treated with or without a glucocorticoid receptor antagonist RU486 (1 µM) or CpdA (1 and 10 µM) for 1 h in BEAS-2B cells, followed by 1.5 h stimulation with NTHi and then the expression of PDE4B mRNA was analyzed by Q-PCR.

### 4.4. Real-Time Quantitative and Semi-Quantitative RT-PCR Analyses

Total RNA was extracted with TRIzol (Life Technologies, Waltham, MA, USA) by following the manufacturer’s instruction. The reverse transcription reaction was performed by using 1 µg of RNA in 20 µL of TaqMan reverse transcription reagents (Life Technologies, Waltham, MA, USA) as described previously [28,58]. For quantitative real-time RT-PCR analysis, PCR amplifications were performed and quantified as described previously [58]. In brief, the reactions were performed in triplicate containing 2xUniversal Master Mix, 1 µL of cDNA, 300 nM primers in a final volume of 12.5 µL and they were analyzed in a 96-well optical reaction plate (Life Technologies, Waltham, MA, USA). Reactions were amplified under the following protocol: 95 °C for 20s followed by 40 cycle of 95 °C for 3 s and 60 °C for 30 s and quantified using StepOnePlus Real-Time PCR System and the manufacturer’s corresponding software (StepOnePlus Software v2.3; Applied Biosystems, Waltham, MA, USA). The primer sequences used are listed in Table 1. The relative quantities of mRNAs were obtained by using the comparative Ct method and were normalized using human *cyclophilin* or mouse *GAPDH* as an endogenous control. For semi-quantitative RT-PCR analysis, PCR amplifications were performed with PrimeSTAR Max polymerase (Takara, Shiga, Japan) by following the manufacturer’s instruction. The primers for human PDE4B2 and GAPDH have been previously described [28].

### 4.5. Plasmids and Transfections

All transient transfections were carried out as described previously [28]. Human PDE4B2 cDNA sequences were generated and inserted into the BamHI and HindIII sites of the pcDNA3.1/mycHis(-) vector. All transient transfections were carried out by using TransIT-LT1 reagent (Mirus, Madison, WI, USA) following the manufacturer’s instruction.

### 4.6. Immunoblotting

Whole-cell lysates were harvested and separated on 10% SDS-PAGE gels, and transferred to polyvinylidene difluoride (PVDF) membrane (GE Healthcare Life Sciences, Freiburg, Germany). Western blot analysis was performed as described previously [58]. The membrane was blocked with 5% non-fat dry milk in Tris-buffered saline (TBS) buffer (50 mM Tris-HCl, 150 mM NaCl, pH 7.5). The membrane was then incubated in a 1:1000 dilution of a primary antibody with 5% BSA in TBS buffer. After five times washes with 0.1% Tween 20 in TBS buffer, the membrane was incubated with 1:5000 dilution of the corresponding secondary antibody with 5% non-fat dry milk in TBS buffer. Samples were visualized by using Amersham ECL Prime Regent (GE Healthcare Life Sciences, Freiburg, Germany).

### 4.7. Mice and Animal Experiments

For NTHi-induced inflammation, we used a well-established C57BL/6J mouse model (8 weeks old female) as previously reported [28,45]. Mice were anaesthetized intraperitoneally with a cocktail of ketamine (70 mg/kg)/xylazine (6 mg/kg) and then intratracheally inoculated with NTHi for 5 h at a concentration of 5 × 10^7^ CFU per mouse and saline was inoculated as control [28]. For inhibition study, mice were pretreated with roflumilast (5 mg/kg), dexamethasone (1 mg/kg), or roflumilast (5 mg/kg) with dexamethasone (1 mg/kg) intraperitoneally 2 h before NTHi inoculation [28,59]. The mice were then sacrificed. For assessing the recruitment of polymorphonuclear neutrophils (PMN) in bronchoalveolar lavage (BAL) fluid, BAL fluid was collected by cannulating the trachea with sterilized PBS in mice followed by staining with Diff-Quik Staining system (Modified Giemsa Staining, St. Louis, MO, USA). All animal studies were carried out in accordance with the guidelines of, and were approved by, the Institutional Animal Care and Use Committee at Georgia State University (A13034, 15 October 2013).

### 4.8. Histology and Immunofluorescence Assay

For histological analysis, we performed hematoxylin and eosin (H&E) staining as described previously [28]. Immunofluorescence detection of PDE4B proteins were performed using rabbit anti-PDE4B and FITC-conjugated goat anti-rabbit IgG (Santa Cruz Biotechnology, Dallas, TX, USA) in the paraffin section of mouse lung tissue. Stained sections were observed under light- or fluorescence-microscopy systems (AxioVert 40 CFL, AxioCam MRC, and AxioVision LE Image system, Carl Zeiss, Oberkochen, Germany). Histopathology was scored as described previously [60].

### 4.9. Statistical Analysis

All experiments were repeated three times with consistent results. Data were shown as mean ± SD. Statistical analysis was assessed by unpaired two-tailed Student’s *t*-test and * *p* < 0.05 was considered statistically significant.

## Figures and Tables

**Table 1:** Primer sequences for quantitative real-time RT-PCR.

Primer Name	Forward (5′-3′)	Reverse (5′-3′)
human *PDE4B*	CTATACCGATCGCATTCAGGTC	CTGTCCATTGCCGATACAATT
human *PDE4B2*	AGCGGTGGTAGCGGTGACTC	GCAGCGTGCAGGCTGTTGTG
human *CXCL1*	TGCAGGGAATTCACCCCAAG	AGCTTTCCGCCCATTCTTGA
human *CXCL2*	GTGTGAAGGTGAAGTCCCCC	AGCTTTCTGCCCATTCTTGA
human *CCL5*	CTACACCAGTGGCAAGTGC	CTTTCGGGTGACAAAGACGAC
human *CCL7*	GGCTTGCTCAGCCAGTTG	GGTGGTCCTTCTGTAGCTCTC
human *Cyclophillin A*	CGGGTCCTGGCATCTTGT	GCAGATGAAAAACTGGGAACCA
mouse *pde4b2*	GTAGAGGCCAGTTCCCATCA	CCAACACCTAGTGCAGAGC
mouse *cxcl1*	CATGGCTGGGATTCACCTCA	CCTCGCGACCATTCTTGAGT
mouse *cxcl2*	TCAATGCCTGAAGACCCTG	GCAAACTTTTTGACCGCCCT
mouse *ccl5*	CCTCACCATATGGCTCGGAC	ACGACTGCAAGATTGGAGCA
mouse *ccl7*	CAATGCATCCACATGCTGCT	GCAGACTTCCATGCCCTTCT
mouse *gapdh*	ACCCAGAAGACTGTGGATGG	GGATGCAGGGATGATGTTCT

## Abbreviations

PDE4B	Phosphodiesterase 4B
NTHi	Nontypeable *Haemophilus influenzae*
GR	Glucocorticoid receptor
COPD	Chronic obstructive pulmonary disease
CF	Cystic fibrosis
GCs	Glucocorticoids
PMN	Polymorphonuclear neutrophils
BAL	Bronchoalveolar lavage
